# Characterization of human Fc alpha receptor transgenic mice: comparison of CD89 expression and antibody-dependent tumor killing between mouse strains

**DOI:** 10.1007/s00262-023-03478-4

**Published:** 2023-06-20

**Authors:** Marjolein C. Stip, J. H. Marco Jansen, Maaike Nederend, Maria Tsioumpekou, Mitchell Evers, Patricia A. Olofsen, Friederike Meyer-Wentrup, Jeanette H. W. Leusen

**Affiliations:** 1 Center for Translational Immunology, UMC Utrecht, Heidelberglaan 100, 3584 CX Utrecht, The Netherlands; 2 Princess Máxima Center for Pediatric Oncology, Heidelberglaan 25, 3584 CS Utrecht, The Netherlands

**Keywords:** Fc alpha receptor, Immunoglobulin A, Neutrophils, Genetically engineered mouse model, Immunotherapy

## Abstract

Since mice do not express a homologue of the human Fc alpha receptor (FcαRI or CD89), a transgenic mouse model was generated in four different backgrounds (C57BL/6, BALB/c, SCID and NXG) expressing the FcαRI under the endogenous human promoter. In this study, we describe previously unknown characteristics of this model, such as the integration site of the *FCAR* gene, the CD89 expression pattern in healthy male and female mice and in tumor-bearing mice, expression of myeloid activation markers and FcγRs and IgA/CD89-mediated tumor killing capacity. In all mouse strains, CD89 expression is highest in neutrophils, intermediate on other myeloid cells such as eosinophils and DC subsets and inducible on, among others, monocytes, macrophages and Kupffer cells. CD89 expression levels are highest in BALB/c and SCID, lower in C57BL/6 and lowest in NXG mice. Additionally, CD89 expression on myeloid cells is increased in tumor-bearing mice across all mouse strains. Using Targeted Locus Amplification, we determined that the hCD89 transgene has integrated in chromosome 4. Furthermore, we established that wildtype and hCD89 transgenic mice have a similar composition and phenotype of immune cells. Finally, IgA-mediated killing of tumor cells is most potent with neutrophils from BALB/c and C57BL/6 and less with neutrophils from SCID and NXG mice. However, when effector cells from whole blood are used, SCID and BALB/c are most efficient, since these strains have a much higher number of neutrophils. Overall, hCD89 transgenic mice provide a very powerful model to test the efficacy of IgA immunotherapy against infectious diseases and cancer.

**Supplementary Information:**

The online version contains supplementary material available at 10.1007/s00262-023-03478-4.

## Introduction

During the last two decades, interest in IgA antibodies and their receptor—the Fc alpha receptor I (FcαRI) or CD89—has increased significantly. Initially, most antibody research was devoted to the four IgG isotypes and their Fc gamma receptors (FcγRs) and currently IgG is still the only isotype used in antibodies for therapeutic applications. However, lately many studies have highlighted the potential of IgA/FcαRI immunotherapy as a treatment for both infectious diseases [1, 2] and cancer [3–6] (reviewed in [7, 8]).

The FcαRI is a member of the immunoglobulin (Ig) gene superfamily, but has only 20% homology to other Fc receptors and in contrast to all other Fc receptor genes, it is not located on chromosome 1 (1q23.3) [9]. The *FCAR* gene encoding FcαRI is located on chromosome 19 (19q13.4) and its promoter is positioned between 59 and 197 bp downstream of the major transcription start site [10, 11]. FcαRI is expressed exclusively on myeloid cells, such as neutrophils, eosinophils, monocytes, macrophages and subsets of dendritic cells (DCs) [12] and can be induced on Kupffer cells as well [13]. Monomeric IgA in complex with antigen or IgA-opsonized target cells can crosslink FcαRI and trigger specific effector functions in myeloid cells, such as antibody-dependent cellular cytotoxicity (ADCC) or phagocytosis (ADCP), antigen presentation and neutrophil extracellular trap (NET) formation [14–16]. In many other species, such as rats and chimpanzees, a homologue of the human FcαRI was found, but not in mice and canines [17–19]. The expression of IgA is different among species as well. Humans and primates express two isotypes, IgA1 and IgA2, and most other mammals (including mice) have only one isotype [20]. Rabbits have the most complex IgA system with up to 15 IgA isotypes [21–23].

Though mice express IgA, they do not express an FcαRI. The FcαRI was lost during evolution in mice due to competition with bacterial decoy proteins for IgA [24, 25]. The absence of a mouse equivalent of human FcαRI has hampered research exploring the potential of IgA immunotherapy in vivo. To study IgA immunotherapy in vivo, several genetic mouse models have been generated to establish expression of human FcαRI in mice. The group of Jianmin Fang developed human CD89 (hCD89) transgenic mice under the murine CD14 promoter, thereby achieving CD89 expression in monocytes, but not in other myeloid cells [26]. Though this model was suitable to answer their research question, it does not reflect the human situation, where other myeloid cell subsets express CD89 as well. The group of Renato Monteiro described a transgenic model with hCD89 under the mouse CD11b promoter, while the group of Adrian Zuercher developed a model using the cre-lox system to insert hCD89 under the mouse Lysozyme M (LysM) promoter [27, 28]. These models are more representative of the human CD89 expression pattern, as CD89 expression is present in multiple myeloid subsets. Additionally, in the model of Adrian Zuercher CD89 expression was highest in neutrophils and low in monocytes, corresponding to both the human and non-human primate expression pattern [27]. However, CD89 expression in murine eosinophils was absent, while it is present on human eosinophils. Finally, a hCD89 transgenic mouse model was developed in the lab of Jan van de Winkel, in which the endogenous human promoter and regulatory elements were transduced along with the sequence encoding hCD89 [29, 30]. The hCD89 promoter is regulated by the highly conserved transcription factors C/EBPα and Ets protein family members [31], which are widely expressed in mice as well. Therefore, this model was expected to result in a CD89 expression pattern more similar to the one found in humans.

Research using this human promoter-regulated CD89 transgenic mouse model has indeed established that CD89 expression in this model is very similar to the human pattern. First, it was confirmed that CD89 expression was present in neutrophils and subsets of monocytes and DCs, but also in activated Kupffer cells [13, 30, 32]. Interestingly, CD89 could be upregulated in neutrophils and macrophages by cytokines such as GM-CSF and TNF-α, which are known to upregulate CD89 expression in human myeloid cells as well. Additionally, hCD89 transgenic mouse neutrophils and macrophages were able to perform phagocytosis and ADCC mediated by IgA and the FcαRI [4]. Many other—sometimes new—characteristics and mechanisms of IgA and the FcαRI were uncovered in these mice, such as an increase in intracellular free calcium levels upon FcαRI crosslinking and the requirement of Mac-1 (CD11b in complex with CD18) for secretory IgA binding to FcαRI [33, 34]. Finally, the in vivo efficacy of IgA immunotherapy has been established for multiple tumor models in hCD89 transgenic mice, for example by targeting EGFR in intraperitoneal and metastatic A431 (epidermoid carcinoma) tumors, GD2 in neuroblastoma mouse models and CD20 in B cell malignancies (unpublished data and [3, 4, 35, 36]).

However, many characteristics of this human promoter-regulated hCD89 transgenic mouse model are not fully elucidated. For instance, in this study we sought an explanation for the fact that homozygous breeding of these transgenic mice is lethal. Therefore, we first determined the integration site of the transgene. Next, as the introduction of CD89 (signaling) could potentially impact immune cell numbers or phenotype, we compared the immune cell composition and phenotype of WT and hCD89 transgenic mice in various strains. Furthermore, most characteristics of this model were previously described for hCD89 mice on the FVB/N background [30] and not within more commonly used strains, such as C57BL/6, BALB/c, SCID and NXG. Since it is known for more than half a century that immune cell composition and phenotype is different between mouse strains and genders [37, 38], we assessed the hCD89 model in these four strains and in both male and female mice. Finally, we compared efficacy of IgA/CD89-mediated ADCC for each mouse strain and studied CD89 expression in tumor models.

## Methods

### Animal experiments

Mice were housed and bred at the animal facility of the Utrecht University (GDL) from 2001 to 2019. From 2019 onwards they were kept at Janvier Labs (France) and transported to the GDL at least 1 week prior to each experiment. Food and water were provided ad libitum and mice were housed in groups under a 12:12 light–dark cycle. Mice were sacrificed by cervical dislocation. Both male and female mice from C57BL/6JRj, BALB/cByJ, CB17-SCID and NXG (NOD.Cg-*Prkdc*^scid^ *Il2rg*^tm1^Wjl/Rj) or NSG (NOD.Cg-*Prkdc*^scid^ *Il2rg*^tm1^Wjl/SzJ) strains were used. Mice in experimental groups were randomized based on weight, age and cage and researchers were single-blinded. Immune composition and CD89 expression data was generated with mice from Charles River (before 2019) and for the remaining experiments mice from Janvier were used (2019 onwards). All mouse experimental procedures were approved by the institute’s animal ethics committee and by the Dutch Central Authority for Scientific Procedures on Animals (CCD, AVD115002016410 and AVD11500202115442).

### Generation of human FcαRI transgenic mice and cross-breeding

**Fig. 1:**
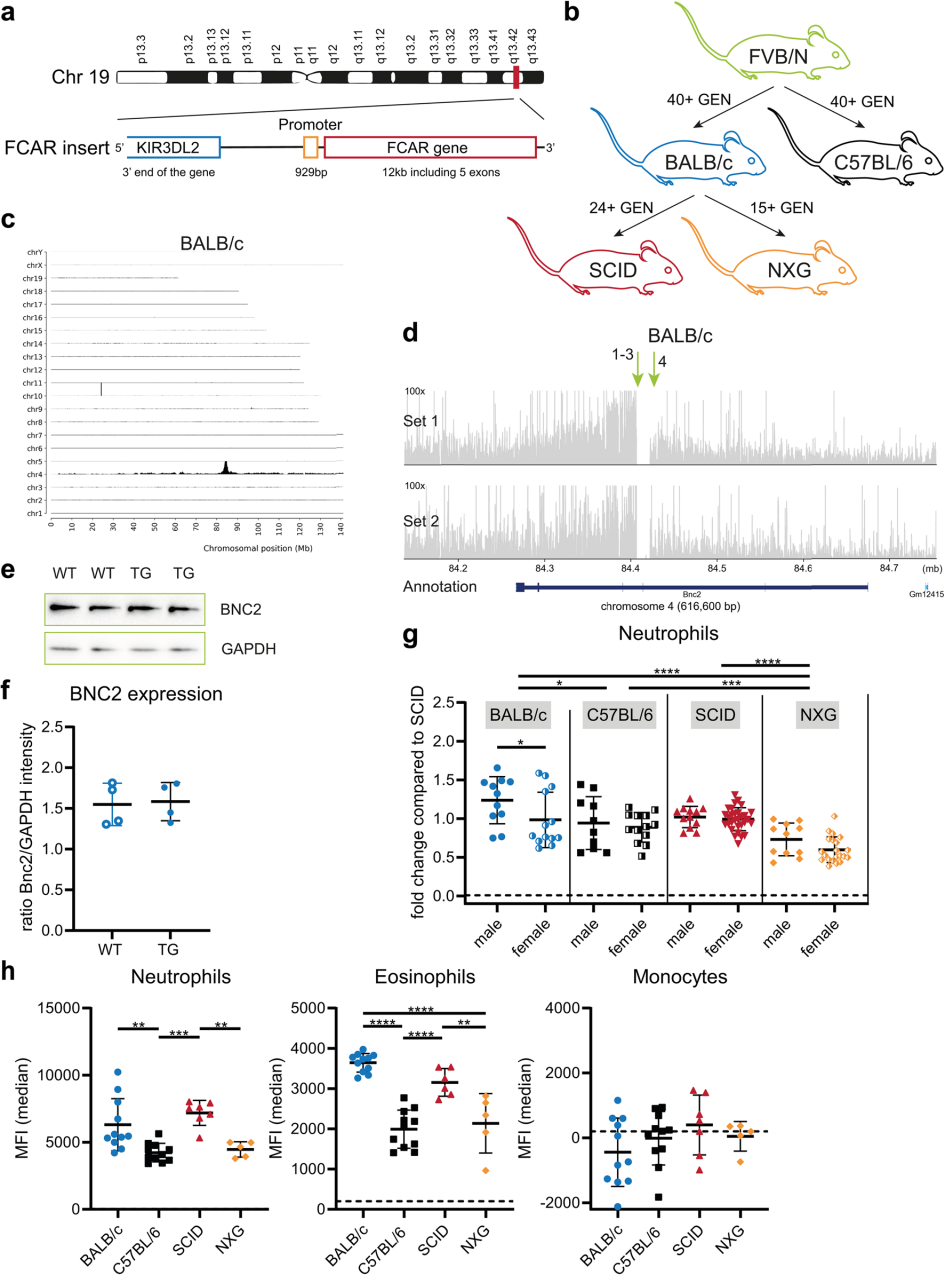
Gene integration site and evaluation of hCD89 expression in circulating myeloid cells. (**A**) Detail of the genomic insert containing the *FCAR* gene that was used to generate the hCD89 mice (**B**) Schematic overview of the order in which hCD89 transgenic mice were generated on 4 different backgrounds. (**C**) Coverage of TLA sequence across the whole mouse genome with primer set 1 in the BALB/c sample. (**D**) Coverage of TLA sequence across the vector integration site in the BALB/c sample. Green arrows indicate the location of breakpoint sequences and genomic rearrangement. (**E**) Representative immunoblot for BNC2 in liver samples from BALB/c mice. Two independent experiments were performed and GAPDH was used as a loading control. (**F**) Normalized values of BNC2 expression in liver samples from BALB/c mice from 2 experiments. (**G**) CD89 expression on circulating neutrophils of both WT and hCD89 TG mice was determined by flow cytometry. Pooled data from at least 9 experiments: data from SCID mice were standardized and data from other strains were related to SCID mice. Means were compared using Two-Way ANOVA with Tukey’s post-hoc test. (**H**) CD89 expression on neutrophils, eosinophils and monocytes from hCD89 TG male mice. Representative experiment of at least 9 other experiments is shown. Means were compared using One-Way ANOVA with Tukey’s post-hoc test. Dotted lines are WT background signal. Data as shown are mean ± SD. GEN = Generations

A 41-kb cosmid clone (R31931) insert of chromosome 19 carrying the 12-kb human *FCAR* gene in a 5.2 kb LAWRIST16 vector was provided by Dr L.K. Ashworth (Human Genome Center, Livermore, CA) [39, 40]. The genomic insert was linearized upon digestion with SfiI restriction enzymes, isolated by electro-elution and injected into fertilized FVB/N oocytes to generate the first generation of transgenic mice [30]. hCD89 transgenic FVB/N mice (founder GG2126) were bred with BALB/c and C57BL/6 mice for at least 40 generations to introduce hCD89 on these backgrounds (Fig. 1A). Subsequently, hCD89 BALB/c mice were bred with SCID and NXG mice for at least 24 and 15 generations respectively. The hCD89 breeding was maintained hemizygously.

### Targeted Locus Amplification, sequencing and alignment

Bone marrow (BM) of hCD89 transgenic BALB/c and NXG mice was isolated by flushing femurs and tibiae with full RPMI medium (RPMI 1640 medium, GlutaMAX (Gibco) supplemented with 10% Fetal Calf Serum (FCS, Bodinco) and 1% penicillin/streptomycin (Gibco)). BM cells were passed through a 70 µm cell strainer, frozen in freezing medium (50% RPMI medium, 40% FCS and 10% dimethyl sulfoxide (DMSO, Sigma)) and transferred to Cergentis for Targeted Locus Amplification (TLA) analysis.

Preparation of the samples and TLA was performed as described by De Vree and colleagues [41]. In short, BM cells were crosslinked using formaldehyde, then DNA was digested with NlaIII. The sample was ligated, crosslinks were reversed and the DNA was purified. To obtain circular chimeric DNA molecules for PCR amplification, DNA molecules were trimmed with NspI and ligated at a DNA concentration of 5 ng/μl to promote intramolecular ligation. As a consequence, a subset of NlaIII (CATG) sites were (re-)digested, generating DNA fragments of approximately 2 kb and allowing the amplification of entire restriction fragments. Primers are listed in Supplemental Table 1. Illumina NGS library was established from the PCR products according to the Nextera DNA Flex Library Prep (Illumina) protocol and libraries were sequenced (paired-end, 2 × 151 bases) on an Illumina sequencer.

Base calling and demultiplexing was performed using The Illumina System, together with the bcl2fastq Conversion Software (Illumina). Using the barcode information, paired-end FASTQ files were generated for each individual amplification of a TLA sample. Reads were mapped to the vector sequences (in this case chromosome 19, human genome hg38 and the LAWRIST16 vector) and host genome (mouse mm10) using BWA-MEM, version 0.7.15-r1140 (settings bwa mem -M -t 4 -B 7 -w 33 -O 5 -E 2 -T 33 -Y) [42]. The resulting mapped BAM files were analyzed using IGV software.

### Western Blot

Liquid nitrogen frozen livers from BALB/c mice were grinded with mortar and pestle. Liver samples were lysed for 60 minutes (min) in RIPA buffer (Sigma) containing a protease/phosphatase inhibitor cocktail (Cell Signaling) and sonicated 3 times for 8 seconds. Lysates were centrifuged twice at 4 °C for 1 hour at 16,000× *g* and supernatants were collected and stored at -80 °C. Protein concentration was determined using a Pierce BCA protein assay kit (ThermoFisher). 40 µg protein was boiled for 5 min in Laemmli sample buffer (Biorad) containing 10 mM dithiothreitol (DTT) and subjected to SDS-PAGE, followed by immunoblotting.

Specifically, proteins were separated by gel electrophoresis using 20% Tris–Glycine gels (ThermoFisher) and electro-transferred to a nitrocellulose membrane using the High MW program on a Trans-Blot Turbo Transfer System (Biorad). Membranes were blocked in 5% skim milk powder diluted in PBS, 0.1% Tween-20 (PBS-T) and incubated over-night at 4 °C with primary antibodies (rabbit anti-mouse BNC2 polyclonal antibody, 1:500 or mouse anti-GAPDH, 1:5,000 (ThermoFisher)). The next day, membranes were washed three times with PBS-T and incubated for 1 hour at room temperature (RT) with horseradish peroxidase-conjugated anti-rabbit or anti-mouse IgG antibodies (ThermoFisher) in PBS-T. Protein bands were visualized using enhanced chemiluminescence (ECL) detection (ThermoFisher) on a charge-coupled device (CCD) camera and Image Lab software was used for analysis and quantification.

### Mouse tumor models

For the Ba/F3 tumor model, 2.5*10^6^ cells Ba/F3 cells were injected subcutaneously on the right flank of BALB/c mice. 9464D-GD2-luc2 cells were generated as described previously [43]. 9464D-GD2 cells were collected in PBS and 0.5*10^6^ cells were injected intraperitoneally in C57BL/6 mice. IMR32 cells were dissolved in a 1:1 mixture of PBS and high concentration matrigel (Corning) and 2.5*10^6^ cells were injected subcutaneously on the right flank of both SCID and NXG mice. After 14 days (Ba/F3 model), 27 days (9464D-GD2-luc2 model) or 51 days (IMR23 model) tumor and blood samples were collected for analysis.

Mouse tumors were carefully excised and collected in ice cold PBS. Tumors were cut and digested using the mouse tumor dissociation kit from Miltenyi. Up to 1 gram of tumor tissue was transferred to C tubes (Miltenyi) containing enzyme mix (DMEM culture medium, 100 μL Enzyme D, 50 μL Enzyme R, and 10 μL Enzyme A) and the 37C_m_TDK_1 program was run on a gentleMACS Octo Dissociator. Following dissociation, tumor cells were filtered using a 100 µm cell strainer and then used for further applications.

### Patient PMN isolation and CD89 staining

Blood was obtained from patients in the UNICIT cohort of the UMC Utrecht. Blood was added on a Ficoll (GE Healthcare)/Histopaque 1119 (Sigma) layer and centrifuged for 20 min at 1500 RPM. Afterwards, low-density polymorphonuclear leukocytes (PMN) and high-density PMN were collected from the interphase between serum and Ficoll or in the Histopaque layer, respectively.

### Antibody staining and flow cytometry

Blood was sampled from the submandibular vein in lithium-heparin tubes (Sarstedt). 30 µL of blood was stained with 30 µL of antibody solution and incubated for 15 min on RT (antibody panels described in Supplemental Tables 2–4). Next, samples were fixated and erythrocytes were lysed using the BD FACS lysing solution for 5–10 min on RT. After washing with PBS, latex beads were added to quantify cell numbers on a Canto II flow cytometer (BD).

Dissociated tumor cells were stained with antibodies for 45 min on 4 °C as in Supplemental Table 5 as well as with TO-PRO3 (1:50,000 ThermoFisher) and analyzed on the LSRFortessa (BD).

Patient PMN were washed in PBS, stained with PE anti-CD89 (BD, clone A59) for 30 min on 4 °C, followed by an additional PBS wash. Patient PMN were analyzed on a Canto II flow cytometer (BD).

### Cell culture

A431, SKBR3, Ba/F3 and 9464D-GD2-luc2 cells were cultured in RMPI-1640, whereas IMR32 cells were cultured in DMEM medium. Both culture media were supplemented with HEPES, Glutamax, 10% heat-inactivated fetal calf serum (FCS, Bodinco) and 100 U/mL penicillin–streptomycin (p/s, Gibco). Neuroblastoma cells (IMR32 and 9464D-GD2-luc2) were additionally supplemented with 2% non-essential amino acids (ThermoFisher) and Ba/F3 cells with 0.1 ng/mL murine IL-3 (Immunotools). All cells were cultured at 37 °C in a humidified incubator containing 5% CO_2_. Cells were not cultured past 20 passages and they were tested every 6 weeks for mycoplasma using a Mycoalert mycoplasma detection kit (Lonza).

### ^51^Cr-release a﻿ssay﻿s

Mice were injected subcutaneously with 20 µg recombinant human PEGylated G-CSF and after 4 days blood was collected in lithium-heparin tubes under terminal anesthesia. Murine neutrophils were isolated from blood by performing erythrocyte lysis (Biolegend) twice for 4 min at 4 °C, followed by magnetic separation using anti-Ly-6G Microbeads (Miltenyi) according to manufacturer’s instructions. Neutrophils were added in an effector-target (E:T) ratio of 40:1 and for Whole Blood (WB) assays 25 µL of whole blood was added per condition.

Target cells were labeled with radioactive chromium-51 (Na_2_^51^CrO_4_, PerkinElmer) for 2 h and washed three times. Antibodies against EGFR (A431), GD2 (IMR32) or HER-2 (SKBR3) were added in concentrations as indicated per experiment. The killing assays were incubated for 4 h at 37 °C in a humidified incubator containing 5% CO_2_. Plates were centrifuged and supernatant was transferred to lumaplates (PerkinElmer) to assess radioactivity induced scintillation (in counts per minute, cpm) on a beta-gamma counter (PerkinElmer). Specific lysis was calculated using the formula: ((Experimental cpm—basal cpm)/(maximal cpm—basal cpm))*100, with maximal lysis determined by incubating target cells with 1.25% triton and minimal lysis determined by chromium release of target cells in the absence of antibodies and effector cells.

### Analysis, statistics and software

Flow cytometry analysis was done in FlowJo (TreeStar). Marker expression and immune cell composition data were pooled from different experiments conducted over a period of approximately 1 year. To compensate for day-to-day variation, data points from SCID mice were first standardized for each experiment and thereafter data from other strains were related to SCID mice.

Statistical analysis was conducted using GraphPad Prism software (version 9.3.0). Means are represented with SD values. Immune cell composition and marker expression were evaluated using Two-Way ANOVAs, first to compare between wildtype and transgenic mice within a strain (when applicable) and next to compare between strains. ADCC assays were compared using Two-Way ANOVAs (or Mixed-Effects model in case of missing data). For all analyses Tukey’s post-hoctest was used. Significance is indicated by * as P < 0.05, ** as P < 0.01, *** as P < 0.001 and **** as P < 0.0001.

## Results

### Breeding and integration site

We first determined the size of the transgene (part of chromosome 19 containing the *FCAR* gene, Fig. 1A) that was integrated in hCD89 transgenic (TG) mice, as well as the integration site and vector copy number using the Targeted Locus Amplification (TLA) protocol of Cergentis [41]. We performed the analysis with BALB/c and NXG mice, since they were the first and last strain respectively to be generated (Fig. 1B). In both mice the sequence of interest was integrated in chromosome 4, accompanied by a complex rearrangement, including the co-integration of a 146 kb genomic fragment from chromosome 10 (Fig. 1C, D and Supplemental Fig. 1A–C). Additionally, 220 bp of the Lawrist16 vector sequence was integrated at the same site. Because of the complexity of the rearrangements and the multiple breakpoint sequences, the exact sequence at the integration site could not be established (Supplemental Table 2). Coverage on the vector-side is 2–5 times higher than on the genome-side of the integration side, hence it is estimated that the copy number of the insert is 2–5. TLA sequencing showed that the *FCAR* gene is the only complete gene on the chromosome 19 insert (Supplemental Fig. 1D). Furthermore, a part of the *KIR3DL2* gene is present, but this gene will not be functional.

Alignment in RefSeq of the vector integration site revealed that vector integration disrupted the mouse basonuclin 2 (*bnc2)* gene. In the past it was noted that homozygous hCD89 transgenic mice were unable to survive beyond 24 h, which corresponds to the phenotype described for *bnc2*^−/−^ mice in literature [44]. To assess BNC2 expression levels in hemizygous hCD89 mice, we performed western blots for BNC2 on liver samples of wildtype (WT) and hCD89 TG mice. Interestingly, both WT and TG mice expressed similar levels of BNC2 in the liver (Fig. 1E, F), indicating that expression of one allele is sufficient to induce normal levels of BNC2.

### In healthy transgenic mice CD89 is expressed by neutrophils and eosinophils, but not monocytes

Next, we characterized CD89 expression in circulating neutrophils and other immune subsets from different mouse strains by analyzing blood samples from healthy male and female, WT and hCD89 TG mice using flow cytometry. CD89 was expressed by neutrophils from all 4 strains, but interestingly CD89 expression levels were highest in BALB/c and SCID, lower in C57BL/6 and lowest in NXG mice (Fig. 1G). Additionally, we observed that CD89 expression was significantly higher in neutrophils from male compared to female BALB/c mice and a similar trend was present in other mouse strains (Fig. 1G).

CD89 expression on eosinophils was generally lower compared to neutrophils (Fig. 1H), corresponding to the human situation. Similar to neutrophils, CD89 expression was highest on eosinophils from BALB/c and SCID and lower on C57BL/6 and NXG eosinophils (Fig. 1H). Surprisingly, CD89 was not detected on circulating monocytes, although it is known to be expressed on healthy human monocytes (Fig. 1H). As expected, CD89 was not expressed on T and B lymphocytes (data not shown).

### Immune cell composition in the circulation is similar between WT and hCD89 TG mice but differs among mouse strains

**Fig. 2:**
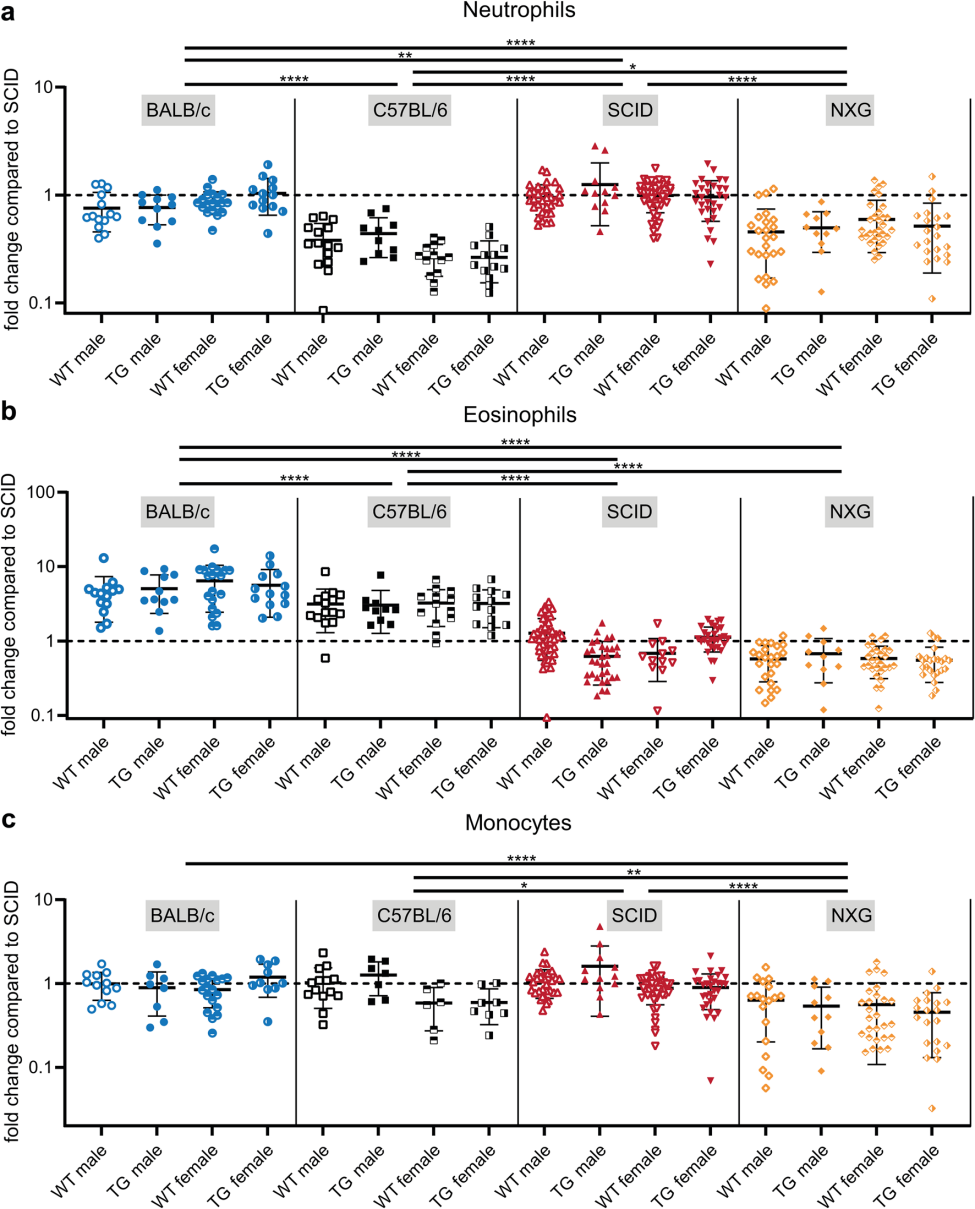
Comparison of myeloid cell numbers in the circulation of WT and hCD89 TG mice. Blood was obtained from both female and male, WT and TG mice from all 4 strains. Antibody staining and subsequent flow cytometry analyses were performed to determine the number of (**A**) neutrophils, (**B**) eosinophils and (**C**) monocytes. Pooled data from at least 9 experiments: data from SCID mice were standardized and data from other strains were related to SCID mice. First, means of mice within a strain were compared and secondly all mice from each strain were pooled and compared using Two-Way ANOVA with Tukey’s post-hoc test

Since we often use both WT and hCD89 TG mice in experiments, we evaluated whether the integration of the transgene affects the circulating leukocyte composition of hCD89 TG mice. Flow cytometry analysis of blood samples from healthy male and female, WT and hCD89 TG mice revealed that there were no differences in total leukocyte, neutrophil, eosinophil, monocyte and T and B cell numbers between WT and hCD89 TG mice in any of the 4 mouse strains (Fig. 2A–C and Supplemental Fig. 2A–C).

However, we did observe many differences in leukocyte composition between mouse strains. As expected, overall leukocyte numbers were higher in immunocompetent BALB/c and C57BL/6 mice compared to immunocompromised SCID and NXG (Supplemental Fig. 2A). Neutrophil numbers were higher in BALB/c and SCID mice compared to C57BL/6 and NXG mice (Fig. 2A). BALB/c mice had more eosinophils compared to C57BL/6 mice, but both of these strains had much more eosinophils compared to immunocompromised mouse strains (Fig. 2B). Monocyte numbers were relatively comparable among strains, only NXG mice had significantly less monocytes (Fig. 2C). Additionally, neutrophil and monocyte numbers appeared to be lower in female compared to male C57BL/6 mice, though not significantly (Fig. 2A, C). Finally, T and B cell numbers in BALB/c and C57BL/6 mice were inverted, BALB/c mice had higher T cell and C57BL/6 higher B cell numbers (Supplemental Fig. 1B, C).

### Phenotype of circulating myeloid cells is not affected by the hCD89 insert but differs among mouse strains

**Fig. 3:**
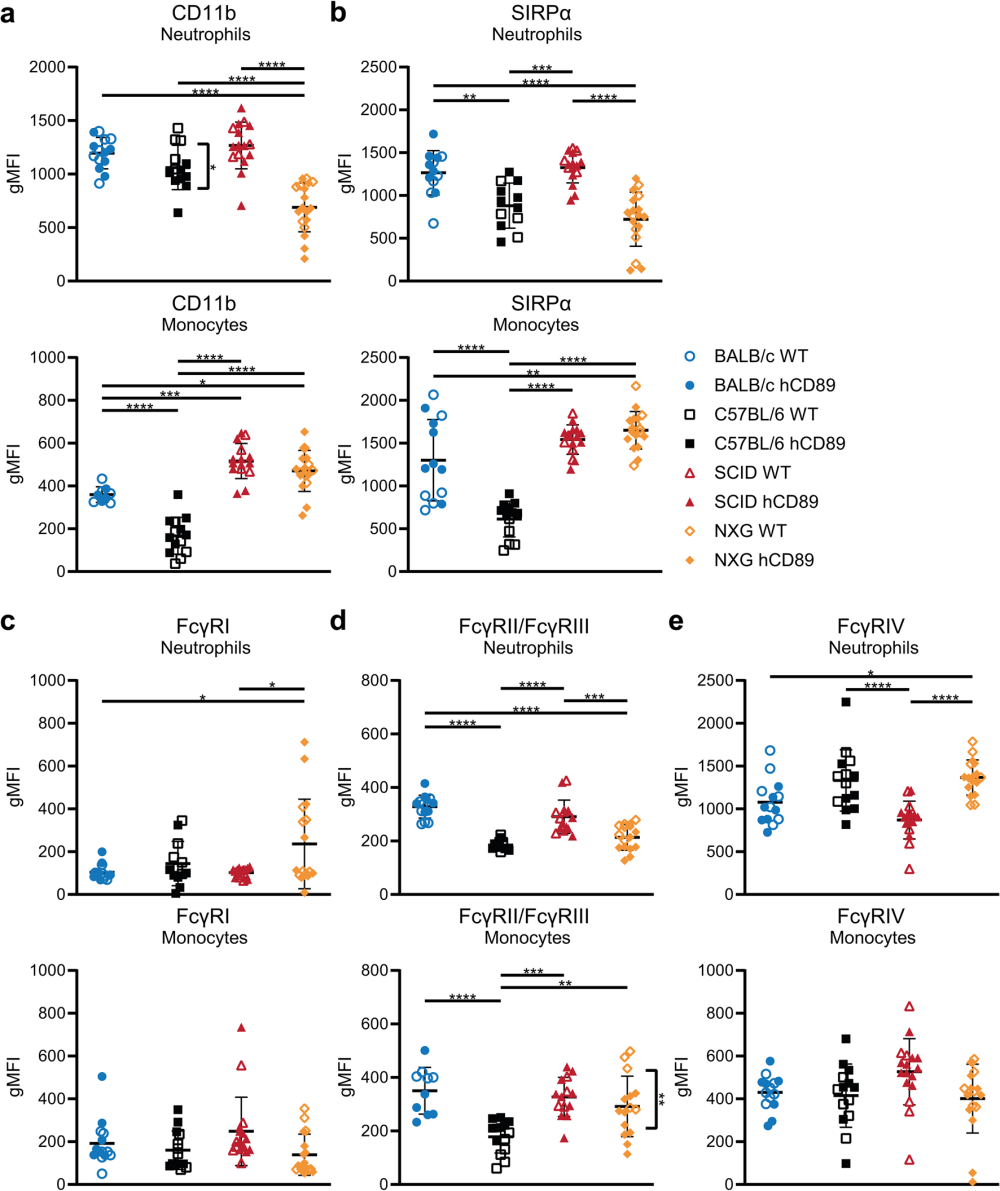
Expression of activation markers and Fc gamma receptors. Blood was obtained from male WT and hCD89 TG mice from all 4 strains. Antibody staining and subsequent flow cytometry analyses were performed to determine expression of (**A**) CD11b, (**B**) SIRPα, (**C**) FcγRI, (**D**) FcγRII/FcγRIII and (**E**) FcγRIV on neutrophils and monocytes. Pooled data from at least 3 experiments: data from SCID mice were standardized and data from other strains were related to SCID mice. First, means of WT and hCD89 TG mice within a strain were compared and secondly all mice from each strain were pooled and compared using Two-Way ANOVA with Tukey’s post-hoc test

Although we did not observe differences in the *number* of circulating leukocytes between WT and hCD89 TG mice, we wanted to study effects of hCD89 transgene integration on the immune cell phenotype as well. Therefore, we first analyzed expression of myeloid activation marker CD11b using flow cytometry. Since it recently became clear that IgA immunotherapy can be significantly enhanced by blocking the CD47/signal-regulatory protein alpha (SIRPα) axis (a myeloid immune checkpoint) [44–47], we studied expression of SIRPα in the hCD89 TG mice as well. Again, no significant differences in CD11b or SIRPα were observed between WT and hCD89 TG mice in any of the 4 strains, except for a small decrease in CD11b expression on C57BL/6 neutrophils (Fig. 3A, B). Interestingly, CD11b expression on NXG neutrophils was rather low compared to neutrophils from other strains, suggesting a low activation status of NXG neutrophils in healthy conditions (Fig. 3A). On the other hand, CD11b expression on monocytes was highest in SCID and NXG, lower in BALB/c and lowest in C57BL/6 mice (Fig. 3A). SIRPα expression on both neutrophils and monocytes from C57BL/6 mice was low compared to other strains, whereas SIRPα in NXG mice was only low on neutrophils, but not on monocytes (Fig. 3B).

When studying the efficacy of IgA antibodies in mouse models, it is often performed in comparison to IgG antibodies. Hence, we studied the expression of receptors for IgG (FcγRs) on circulating myeloid cells of hCD89 TG mice as well. No changes in FcγR expression were found between WT and hCD89 TG mice, aside from a small decrease in FcγRII/FcγRIII expression on NXG monocytes (Fig. 3C–E). In accordance with literature [48], expression of the high affinity, activating IgG receptor FcγRI was absent on neutrophils and monocytes (Fig. 3C), whereas FcγRII and FcγRIII (inhibitory and activating receptors respectively) were modestly expressed (Fig. 3D). Compared to the other strains, FcγRII/FcγRIII was less expressed in neutrophils and monocytes from C57BL/6 mice and in neutrophils from NXG mice. FcγRIV was highly expressed in neutrophils from all strains, though expression was highest in C57BL/6 and NXG mice (Fig. 3E). We did not observe high FcγRIV expression in monocytes, since we only selected Ly6C^high^ monocytes and FcγRIV is known to be mostly expressed in Ly6C^int^ CD115^+^ instead of Ly6C^high^ monocytes.

Subsequentially, we analyzed these surface markers in G-CSF stimulated mice, since G-CSF is often administered in mouse models to activate neutrophils and other myeloid cells and to recruit them to the circulation. Four days after G-CSF stimulation CD89 expression in BM-derived neutrophils was increased, but not in circulating neutrophils (Supplemental Fig. 3A). CD11b expression was reduced in neutrophils and SIRPα expression was unchanged after G-CSF stimulation (Supplemental Fig. 3B, C). Additionally, G-CSF influenced FcγR expression (Supplemental Fig. 4A–C), since FcγRII/FcγRIII expression was enhanced in all myeloid cells. FcγRIV expression was elevated in neutrophils and circulating eosinophils and monocytes.

### Comparison of ADCC capability between strains

**Fig. 4:**
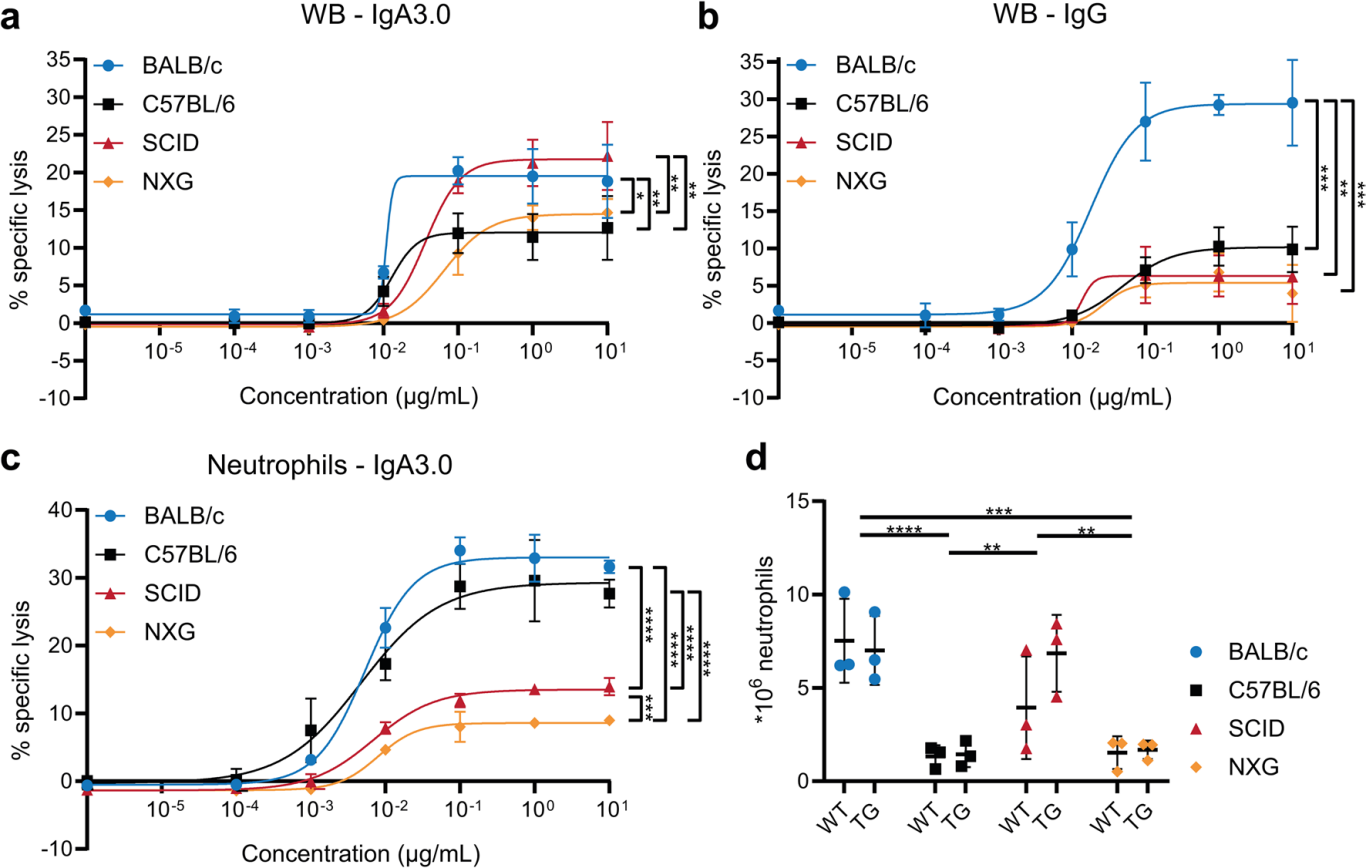
Comparison of IgA- and IgG-mediated ADCC capacity of 4 different mouse strains. ^51^Cr-release assays against EGFR-expressing A431 cells comparing (**A**) IgA3.0- or (**B**) IgG-mediated ADCC with whole blood from 4 different TG mouse strains. (**C**) ^51^Cr-release assays showing concentration-dependent ADCC of EGFR-expressing A431 cells after binding of anti-EGFR IgA antibodies by isolated neutrophils from 4 different hCD89 TG mouse strains. ADCC capacity of strains was compared using a RM Two-Way ANOVA with Tukey’s post-hoc test. (**D**) Total number of neutrophils per mouse after Ly-6G MACS isolation. Means were compared using Two-Way ANOVA with Tukey’s post-hoc test. Graphs are representative data of at least 3 different experiments. WB = Whole Blood, WT = wildtype, TG = transgenic

Besides the phenotypic characterization of hCD89 transgenic mice, we compared the functionality of CD89 expressed in different mouse strains by performing antigen-dependent cellular cytotoxicity (ADCC) assays. Since neutrophil numbers are very low in the circulation (Supplemental Fig. 5A), mice were injected with G-CSF 4 days prior to the experiment to mobilize myeloid cells from the bone marrow. Using effector cells from whole blood (WB), IgA-mediated killing of A431 cells, an epidermoid carcinoma cell line, was most efficient by SCID and BALB/c, but less efficient by NXG and C57BL/6 effector cells (Fig. 4A). WB effector cells from BALB/c mice were able to adequately eliminate A431 tumor cells upon IgG stimulation, whereas WB effector cells from other strains did not achieve more than 10% specific lysis (Fig. 4B).

Thereafter, we isolated neutrophils using magnetic-activated cell sorting (MACS) (Supplemental Fig. 5B) to perform ADCC assays with pure neutrophils as effector cells. Interestingly, neutrophils from immunocompetent mouse strains (BALB/c and C57BL/6) induced more tumor cell lysis compared to neutrophils from immunocompromised mice (SCID and NXG) and NXG neutrophils performed even worse than neutrophils from SCID mice (Fig. 4C). A similar pattern was observed when other tumor cells such as SKBR3 (breast cancer) and IMR32 (neuroblastoma) were used as target cells, though these cell lines were more resistant to killing in general (Supplemental Fig. 5C). IgG-mediated killing of these tumor cells by neutrophils was lower than IgA-mediated killing (Supplemental Fig. 5C). Similar to IgA, IgG antibodies induced more tumor cell lysis with neutrophils from immunocompetent strains. When neutrophils from wildtype mice were used as effector cells, only IgG induced tumor cell killing and not IgA, indicating that IgA-mediated ADCC is dependent on the expression of FcαRI (Supplemental Fig. 5D, E).

In both unstimulated (Fig. 1G) as well as G-CSF stimulated mice (Fig. 4D), neutrophil numbers are highest in BALB/c and SCID mice. This is consistent with the higher killing percentages observed in these strains, when equal volumes of whole blood are used in ADCC assays. However, in ADCC assays with equal numbers of neutrophils, neutrophils from immunocompetent mice induce most tumor cell lysis, suggesting that neutrophils from BALB/c and C57BL/6 mice are more potent.

### CD89 expression is elevated in tumor-bearing mice and in PMN-MDSC from cancer patients

**Fig. 5:**
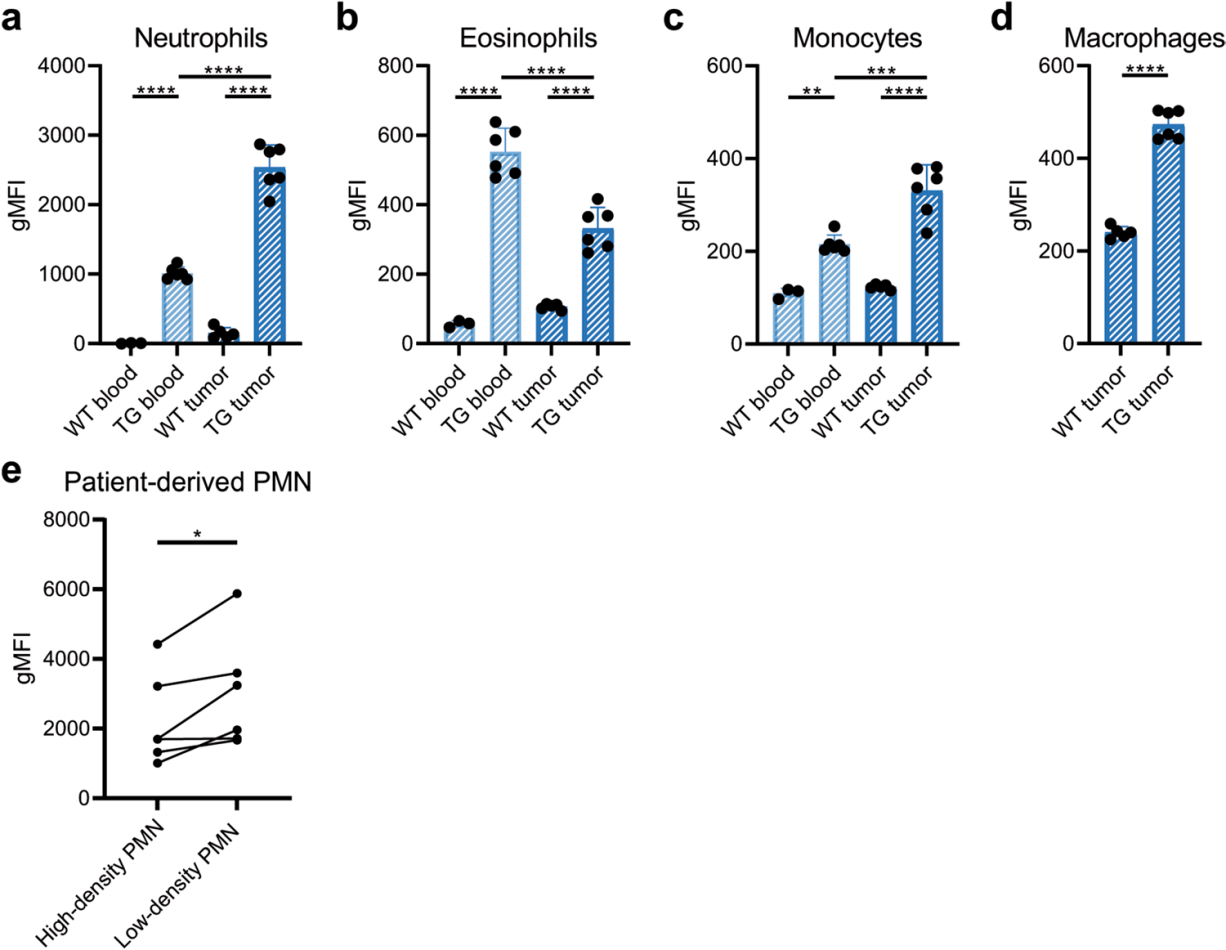
CD89 expression on myeloid cells in the tumor microenvironment. Subcutaneous Ba/F3 tumors in BALB/c mice were established by injecting 2.5*10^6^ Ba/F3 cells subcutaneously. CD89 expression on myeloid cell subsets in the circulation and tumor was determined on day 14 by flow cytometry. Means of (**A**) neutrophils, (**B**) eosinophils and (**C**) monocytes were compared using a One-Way ANOVA with Tukey’s post-hoc test and means of (**D**) macrophages were compared using an unpaired t-test. (**E**) Using flow cytometry, CD89 expression was compared between high-density and low-density PMN of solid cancer patients. Means were compared using a paired t-test. WT = wildtype, TG = transgenic, PMN = polymorphonuclear leukocytes

Finally, we studied CD89 expression in tumor-bearing mice, since IgA research is often performed in the context of mouse tumor models and not in healthy mice. Both WT and hCD89 TG BALB/c mice were injected subcutaneously with 2.5*10^6^ Ba/F3 cells, a murine pro-B cell line, and blood and tumor samples were analyzed after 14 days by flow cytometry. In these tumor-bearing mice, CD89 expression was more than doubled on intratumoral neutrophils compared to circulating neutrophils (Fig. 5A). However, for intratumoral eosinophils we observed the opposite effect: they had lower CD89 expression compared to circulating eosinophils (Fig. 5B). Additionally, CD89 was expressed on monocytes and macrophages in the tumor microenvironment (Fig. 5C-D). Circulating monocytes in tumor-bearing mice started to express CD89 as well (Fig. 5C), whereas circulating monocytes in healthy mice were generally negative for CD89 (Fig. 1H).

Mouse tumor models were established as well for the other mouse strains. Specifically, C57BL/6 mice were injected intraperitoneally with 9464D-GD2 neuroblastoma tumor cells and subcutaneous IMR32 human neuroblastoma xenograft tumors were established in both SCID and NXG mice. In agreement with our data from the BALB/c tumor-bearing mice, in all three models CD89 expression was increased on intratumoral neutrophils, monocytes and macrophages, but decreased on intratumoral eosinophils (Supplemental Fig. 6).

Finally, we related these mouse CD89 expression data to CD89 expression in high-density and low-density neutrophils from solid cancer patients. High-density neutrophils are considered ‘regular’ neutrophils and low-density neutrophils are considered myeloid-derived suppressor cells (PMN-MDSC) that are educated by tumor cells [49]. Using antibody staining and flow cytometry we observed that CD89 expression is higher on low-density compared to high-density neutrophils (Fig. 5E), supporting the findings in mice that CD89 expression is generally increased on tumor-associated myeloid cells.

## Discussion

In this study we thoroughly characterized a hCD89 transgenic mouse model regulated by the endogenous human promoter. Our results, along with previous studies, indicate that CD89 expression and function in this model highly resemble the human situation [12, 46]. We and others observed that CD89 expression is highest in neutrophils, intermediate on other myeloid cells such as eosinophils and DC subsets and inducible on monocytes and Kupffer cells, among others. Interestingly, CD89 expression was increased in all myeloid cells except eosinophils in both blood and tumor samples of tumor-bearing hCD89 transgenic mice. We recently observed that PMN myeloid-derived suppressor cells (PMN-MDSCs) from several solid cancer patients (Fig. 5E), melanoma and colorectal cancer patients have elevated CD89 expression as well (unpublished data). This highlights again the similarity of CD89 regulation between humans and our hCD89 transgenic mice. Furthermore, the fact that CD89 expression is increased on tumor-infiltrating myeloid cells is promising for IgA immunotherapy and remains an interesting topic for future investigation.

Currently, the mice from our hCD89 transgenic model under the endogenous promoter are bred hemizygously. In the past, homozygous breeding of the hCD89 transgenic mice was attempted, but was never successful. We now show that this is caused by the integration site of the *FCAR* gene in our model, leading to disruption of the *bnc2* gene and not due to the introduction of hCD89 itself. BNC2 is an extremely conserved zinc finger protein and is required during embryonal development and the development of germ cells. *Bnc2*^−/−^ mice are born with a cleft palate and abnormalities of craniofacial bones and tongue and most of these mice die within 24 h after birth [43, 47]. This phenotype corresponds to the one observed when homozygous breeding of our hCD89 mice was attempted. However, when maintained hemizygously, hCD89 transgenic mice are fully healthy, able to reproduce and expressing equal levels of BNC2 in the liver as WT mice, indicating that one allele of *bnc2* is sufficient.

Furthermore, we compared the immune cell composition and expression of CD11b, SIRPα and several FcγRs between WT and hCD89 TG mice, and did not find any differences. Hence, we believe that we can use WT littermates as control mice for hCD89 transgenic mice in the future, to reduce unnecessary breeding. On the other hand, we did find major differences in immune cell composition, marker expression and ADCC capacity between mouse strains (Table 1). In the future, these differences between mouse models should be taken into consideration when choosing a model for (IgA) research. For example, when BALB/c mice are chosen, IgA efficacy could be higher compared to C57BL/6, SCID and NXG mice, since BALB/c mice have higher neutrophil numbers and higher CD89 expression, resulting in more ADCC. Additionally, neutrophil infiltration and activation in C57BL/6 has been reported to be limited compared to BALB/c mice in for example lung inflammation models [52]. In many studies, IgA immunotherapy is compared to IgG immunotherapy, since IgG is currently the gold standard. IgA immunotherapy could be relatively more favorable than IgG in BALB/c and SCID mice compared to C57BL/6 and NXG, mice, since the latter two have higher FcγRIV expression, required for IgG effector function. These examples highlight that depending on the mouse model, IgA studies could have different outcomes.

**Table 1:** Comparison of different mouse strains for CD89 expression, myeloid cell numbers and ADCC efficacy

	BALB/c	C57BL/6	SCID	NXG
CD89 expression	++++	++	+++	+
Neutrophil numbers	++++	+	+++	+
Eosinophil numbers	+++	+++	+	+
Monocyte numbers	++	++	++	++
Whole blood ADCC—IgA	++++	+++	+++	+
Whole blood ADCC—IgG	+++	+	+	+
Neutrophil ADCC—IgA	++++	++++	++	+

Additionally, we observed that male hCD89 transgenic mice tend to have higher CD89 expression than females. To our knowledge it is not well described how this translates to the human situation. However, it is known that there are major gender differences in for example neutrophil activation by type I interferons or immunoglobulin levels, including IgA [52–54]. It will be interesting to investigate gender differences in IgA and CD89 expression further in the human setting, but this is beyond the scope of this paper.

In this study, we found that neutrophils in SCID, but especially in NXG mice are much less activated than neutrophils from immunocompetent strains. It has been described that other innate cells such as macrophages and dendritic cells are dysfunctional in NXG (or NSG) mice, even though they are not directly modified by the strain’s specific null mutations (*Prkdc*, *Il2rg*) [56]. Neutrophils from NXG mice could possibly be inactive due to the lack of signaling via IL-2 receptor gamma chain (IL-2rγ) as well, although this has not been described previously. Another possible explanation could lie in the fact that NXG mice have a SIRPα polymorphism, resulting in higher affinity for its target, CD47 [57]. Since the SIRPα/CD47 axis is a myeloid checkpoint, this could explain why IgA-stimulated neutrophils from NXG mice are not able to kill tumor cells properly. Furthermore, we observed that in general neutrophils from all mouse strains induce less ADCC upon IgA stimulation than human neutrophils. Additionally, it is known that neutrophil numbers are lower in mice (~ 10% of circulating leukocytes) than in humans (~ 50–70%). BALB/c mice might yet be the most representative model of the human situation in this regard, since they have the highest neutrophil numbers and most activated neutrophils of all strains evaluated. Possibly the difference between mice and humans is the result of housing under germ-free conditions, resulting in less priming by cytokines and therefore less neutrophil activation in mice. For example, it was established that the gut microbiome is involved in neutrophil aging and activation [58], indicating a relationship between exposure to microorganisms and neutrophil activation. Additionally, it is known that ADCC by mouse neutrophils can be enhanced by activating cytokines such as GM-CSF and TNF-α, further supporting the hypothesis that mouse neutrophils are less activated than human neutrophils in general [30].

Overall, the hCD89 transgenic mouse model provides a very powerful tool to test the efficacy of IgA immunotherapy against infectious diseases and cancer. In this study we thoroughly characterized previously unknown features of this model, such as the integration site of the *FCAR* gene, CD89 expression in healthy and tumor-bearing mice, expression of myeloid activation markers and FcγRs and tumor killing capacity. These data will form a solid base for deciding which model and which mouse strain to choose for in vivo experiments evaluating IgA immunotherapy.

## Supplementary Information

Below is the link to the electronic supplementary material.

Supplementary file1 (PDF 1407 KB)

## Data Availability

Data and unique/stable reagents generated in this study are available from the lead contact with a completed materials transfer agreement. Further information and reasonable requests for resources and reagents will be provided and can be directed to Dr. Jeanette Leusen, j.h.w.leusen@umcutrecht.nl.
