# Supplementary material for: Characterization of human Fc alpha receptor transgenic mice: comparison of CD89 expression and antibody-dependent tumor killing between mouse strains
Source: Cancer Immunol Immunother. 2023 Jun 20;72(9):3063–77. doi: 10.1007/s00262-023-03478-4 (PMC10412663; doi:10.1007/s00262-023-03478-4)
Supplement: Supplementary file 1 — Supplementary file1 (PDF 1407 KB) [file 262_2023_3478_MOESM1_ESM.pdf]

Supplemental Table 1

Primer set	Name/VP	Direction	Binding position		Sequence
			FCAR-chr19	*Lawrist16*	
**1**	set1	RV	2,357	-	ATTCCAGCCTCTAAGTCTCT
	set1	FW	2,713	-	GATCACAAGGTCAGGAGATC
**2**	set2	RV	13,021	-	GGACTCGATTTGGCAGATAT
	set2	FW	13,172	-	CTGATCCTGAGTTCGTCATT

Table S1 | Primers used in TLA analysis

Supplemental Table 2

Marker	Fluorochrome	Clone	Dilution	Company
**Human CD89**	PE	A59	1:50	BD
**CD45**	BV510	30-F11	1:200	Biolegend
**Ly6C**	APC-Cy7	HK1.4	1:200	Biolegend
**Ly6G**	PE-Cy7	1A8	1:400	Biolegend
**Siglec F (CD170)**	BV421	S17007L	1:100	Biolegend
**CD3e**	APC	145-2C11	1:200	eBioscience
**B220**	FITC	RA3-6B2	1:100	Biolegend

Table S2 | Panel of fluorophore-conjugated antibodies for analysis of CD89 expression and phenotyping of circulating immune cells with flow cytometry

Supplemental Table 3

Marker	Fluorochrome	Clone	Dilution	Company
**CD45**	BV510	30F11	1:200	Biolegend
**CD11b**	FITC	M1/70	1:50	BD
**Ly6C**	APC-Cy7	HK1.4	1:200	Biolegend
**Ly6G**	PE	1A8	1:400	Biolegend
**Siglec F (CD170)**	BV421	S17007L	1:100	Biolegend
**SIRPα (CD172a)**	PerCP/Cy5.5	P84	1:50	Biolegend

Table S3 | Panel of fluorophore-conjugated antibodies for analysis of CD11b and SIRPα expression with flow cytometry

Supplemental Table 4

Marker	Fluorochrome	Clone	Dilution	Company
**CD45**	BV711	30F11	1:200	Biolegend
**Ly6C**	PerCP-Cy5.5	HK1.4	1:100	Biolegend
**Ly6G**	PE-Cy7	1A8	1:400	Biolegend
**Siglec F (CD170)**	BV421	S17007L	1:100	Biolegend
**mFcγRI**	PE	X54-5/7.1	1:50	Biolegend
**mFcγRII/III**	FITC	2.4G2	1:100	BD
**mFcγRIV**	BV511	9E9	1:100	Biolegend

Table S4 | Panel of fluorophore-conjugated antibodies for analysis of FcγR expression with flow cytometry

Supplemental Table 5

Marker	Fluorochrome	Clone	Dilution	Company
**CD45**	BV510	30F11	1:200	Biolegend
**CD11b**	FITC	M1/70	1:50	BD
**F4/80**	BV781	BM8	1:200	Biolegend
**Human CD89**	PE	A59	1:50	BD
**Ly6C**	PerCP-Cy5.5	HK1.4	1:100	Biolegend
**Ly6G**	PE-Cy7	1A8	1:200	Biolegend
**Siglec F (CD170)**	BV421	S17007L	1:50	Biolegend

Table S5 | Panel of fluorophore-conjugated antibodies for tumor analysis with flow cytometry

Supplemental Table 6

Breakpoint	Order	Sequence
1	chr10:24,031,978 (head) fused to hg38 chr19:54,852,450-54,852,551, fused to chr4:84,405,464 (head)	AAAGGAAAAATCTACCAAGAAGAGTTCTCAGTTCTGAACATCTATGCTTCAA ATGCAAGGGCACCCACATTCATAAAAGAAACTTAACTAAAGCTCAAAGCAC ACAATGATGGCTACATTGTAAACCCTGGAGCCTGTGACTATTTATGTTATAG GGCAGGGGACTGAAGGGGAAGGTGGAGCTCAGGTTGTTGATGAGTTGAC CTTGATACAACTTTAGAAATTTTTCTTAGATCTAACAGAGATGCTGGTATGTA ACTAGACAGAATACTTCTTACTGATAATTTTACAACCTAGTTTTTTTTAAGAC AGGCTCTCCAAAAT
2	chr4:84,406,787 (tail) fused to Lawrist16-vector Vector:544-615 fused to FCAR-chr19 Vector: 19,982 (tail)	CACAGCCAAGATAAACCGGTACAAAACTCCACTCTGAAGCACACCCCCTTCC ACACTGCACTTTGAAAGACTCTGTTTTTGTCTCTGAGATGGGGTCTCAAGTA TTCCAGGCTGTGCTCAAACTCACCATTAAGCGGGCTAATTCGCCTCGAGGTG GCTTATCGAAATTAATACGACTCACTATAGGGAGACCCAAGCTTAGGATCAC CCTGAGGTCAGAAGTTTGTGACCAGCTTGGCCAACATGGCGAAACCCGTC
3	chr4:84,404,993 (tail) fused to hg38 chr19:54,852,578 (head)	TGTCCCCTCCTGGCTCCCTGGCACAGTAGGAGGGTGGCTTCACAGGGAG ACTTGCCTCCTGTAGGAGACACTCAGTGGGGAATTGCTCCTCAGGCATG AACAGGGTCCTGCTGGGCTCAGTGTAATCACAAGGGTCCGCGTGAGAG GTGGAGGAAGAGGGGAGTGGGGATTAGAGCAGTGTAGTGGGAGGGA GACGCTATCAGCCACTGTGGGCTTTGAAGG
4	chr10:24,031,972 (tail) fused to chr4:84,405,000-84,405,416 fused to chr4:84,422,506 (head)	GTTAGATCCATTTGGTTCATAACTTCTGTTAATTTCACAGTGTCTCTGTTT AGTTTCTGTTTCTATGATCTGTCCATTGCTGAGAGTGGGATGTTGAAGT CTCCCAATATTATTGTGTGGGGTGCACAGGAAAGGCCACACAAGTCCC ATACTGTGGAAATGCTACAATGGGAAGCTCTAGGGAGACTTCACAGTG ACCAAAGAGAGACTAGAGCCTGAACTGAGGCTTTCTGGTTGTCCGATC AGTTCTAACCACTCTGCTAGTAATCCATTTTCAACCTAAGACTGGGTTAT CTAAGTTTATGTTATGCACAGATAGCCTATCACAGCATGGCTATAGAAA GAGGATTCTAGAGTTTGGCCTTGAACTAACTATGAGAGGAAGGATGAC CTTGAACAATCGATCCTCCAGCTTCTACCTCCTGAGCACAGGAATTGTA GACTTGCCCTGCTTTATGTGGTGTTGGGGAATCTAGTACTTTGTGCAGG GCAAGCAAGACCAGTACCAGCTGAACTACAGCCCCAGGCCTCAACATA CTTTTTAAAAAAAACTACTTCAGTCTAGAAACTAATTAAGTGTGGGGGA GGAAGCAAAGAGAGAAGCCCCTAAACTTACGGGTGTATCATTATACAT GACTCTGAATATTCCTATGTCTGTGTGAATACCTAAACAGGGAG

Table S6 | Breakpoint sequences found in BALB/c and NXG mice using TLA analysis. Sequences of human chromosome 19 (including the hCD89 insert) are depicted in red, Lawrist16 vector sequences in orange, mouse chromosome 4 in blue, mouse chromosome 10 in green and homologous bases are in purple.

Supplemental Figure 1

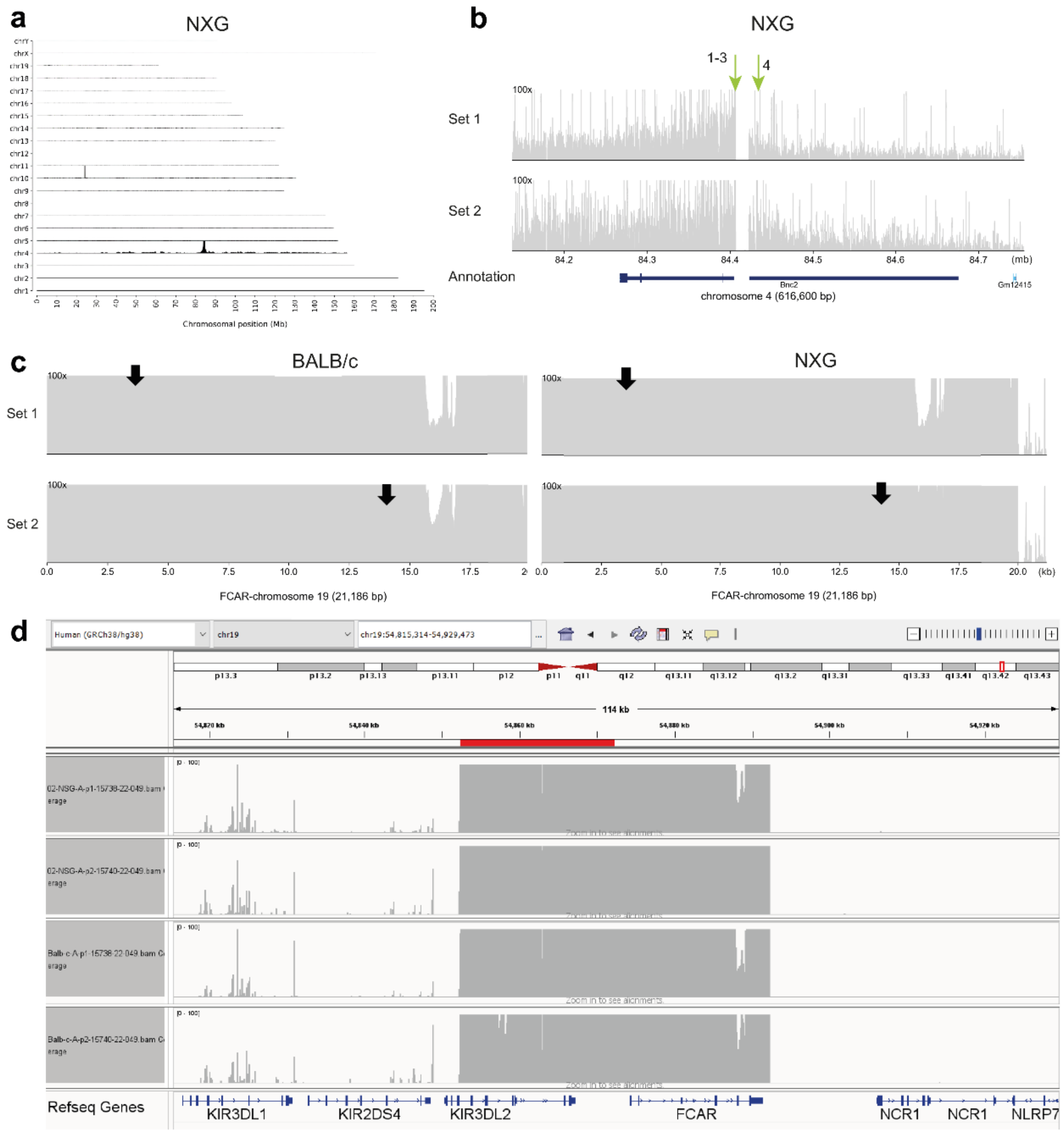

Fig. S1 Determination of gene integration site and copy number (**A**) Coverage of TLA sequence across the whole mouse genome with primer set 1 in the NXG sample. (**B**) Coverage of TLA sequence across the vector integration site in the NXG sample. Green arrows indicate the location of breakpoint sequences and genomic rearrangement. (**C**) Coverage of Next Generation Sequencing across the hCD89 insert for both BALB/c and NXG samples. (**D**) TLA sequence coverage across the human chromosome 19. The green bar represents the human *FCAR* gene, the red bar additional integrated sequences of chromosome 19. The orange arrow indicates the breakpoint with the LAWRIST16 vector.

Supplemental Figure 2

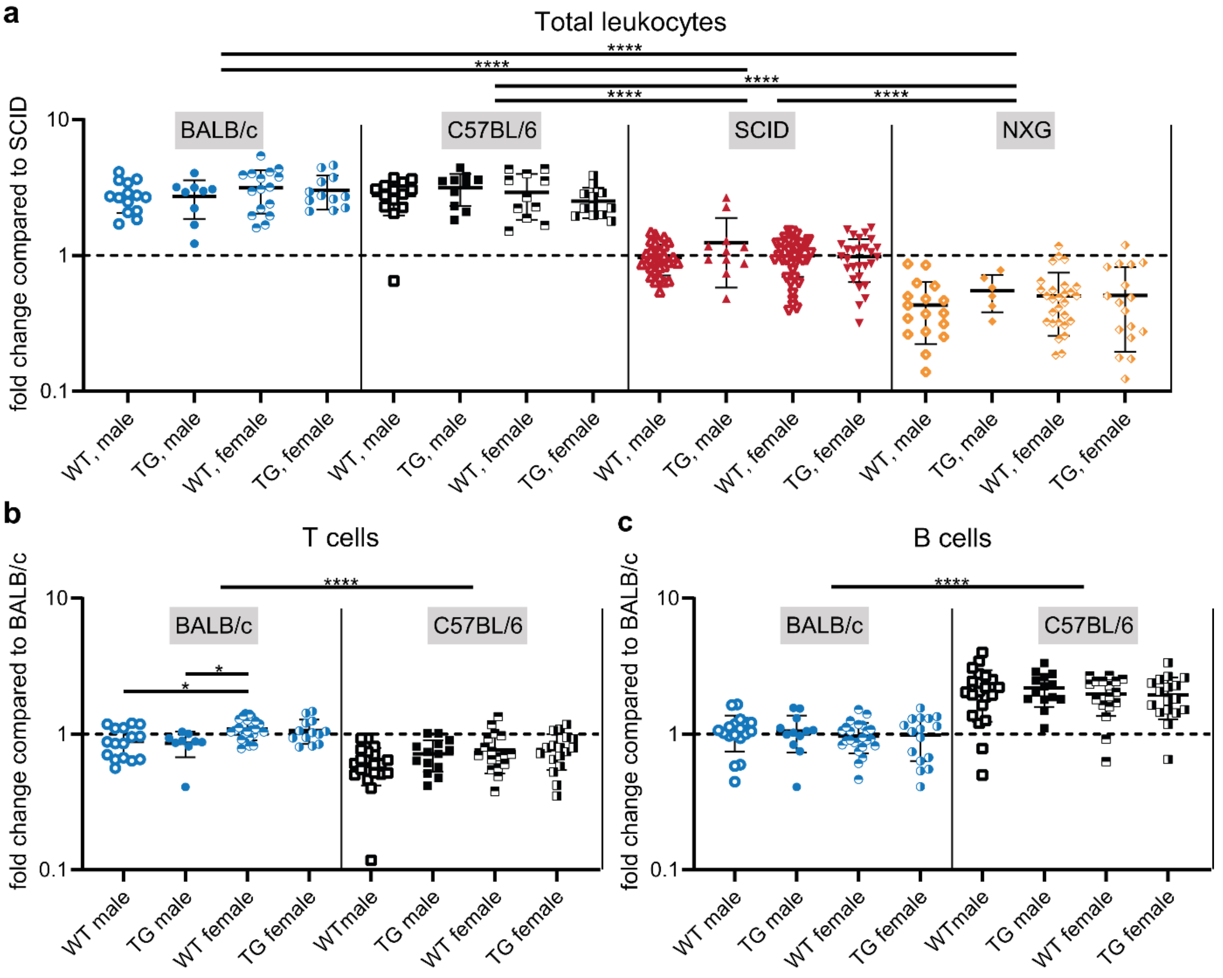

Fig. S2 Comparison of total leukocyte and T and B cell numbers in the circulation of WT and hCD89 TG mice Blood was obtained from both female and male, WT and hCD89 TG mice from all 4 strains. Antibody staining and subsequent flow cytometry analyses were performed to determine the number of (**A**) total leukocytes, (**B**) T cells and (**C**) B cells. Pooled data from at least 9 experiments. For total leukocytes, data from SCID mice were standardized and data from other strains was related to SCID mice. For T and B cells data was related to BALB/c mice. First, means of mice within a strain were compared and secondly all mice from each strain were pooled and compared using Two-Way ANOVA with Tukey's post-hoc test.

Supplemental Figure 3

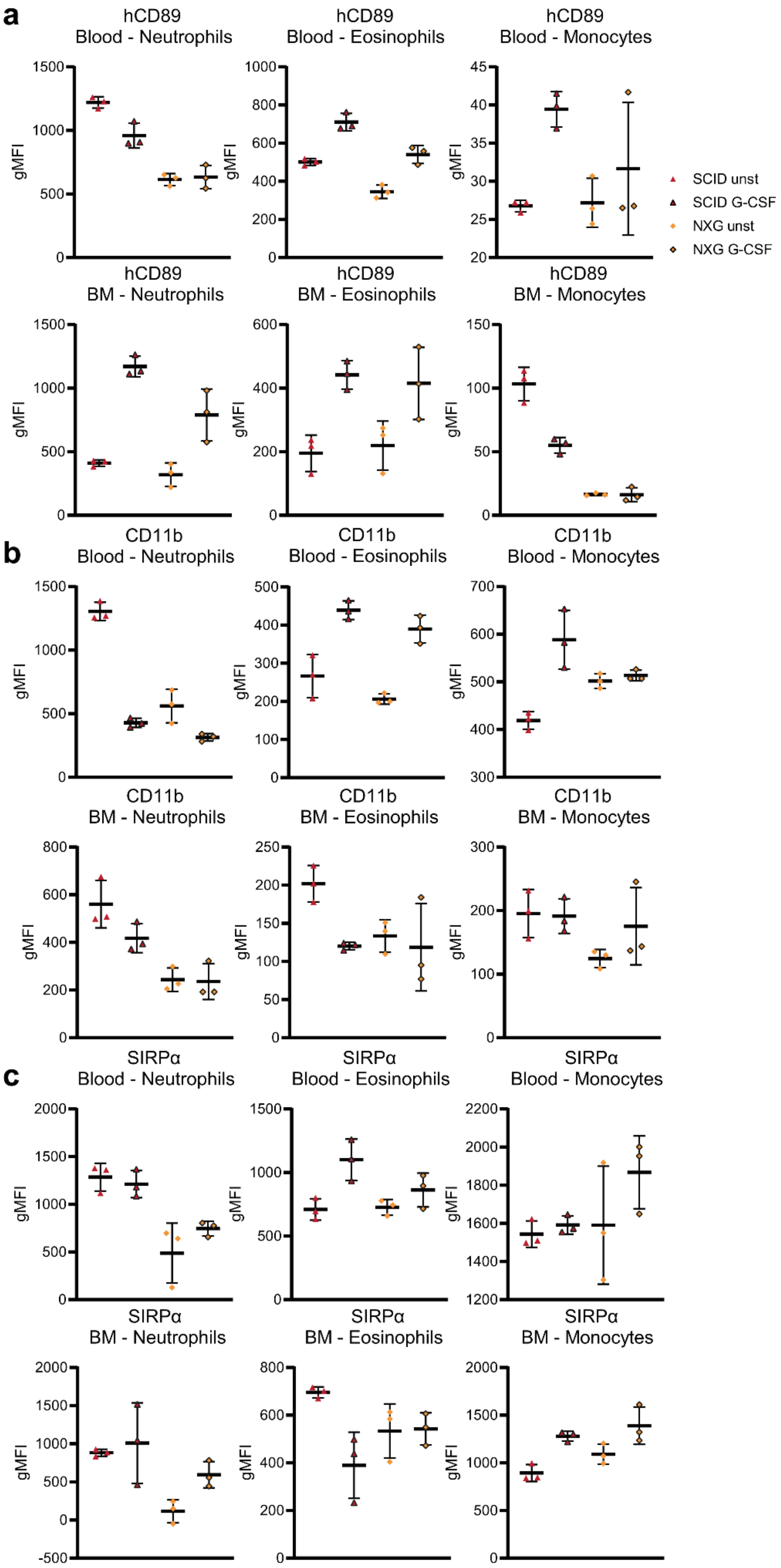

Fig. S3 CD89, CD11b and SIRPα expression in myeloid cells in peripheral blood and bone marrow Blood and bone marrow was obtained from either PEG-G-CSF or unstimulated male hCD89 TG mice from 4 mouse strains. Antibody staining and subsequent flow cytometry analyses were performed to determine expression of (**A**) CD89, (**B**) CD11b and (**C**) SIRPα on neutrophils, eosinophils and monocytes. Unst = unstimulated.

Supplemental Figure 4

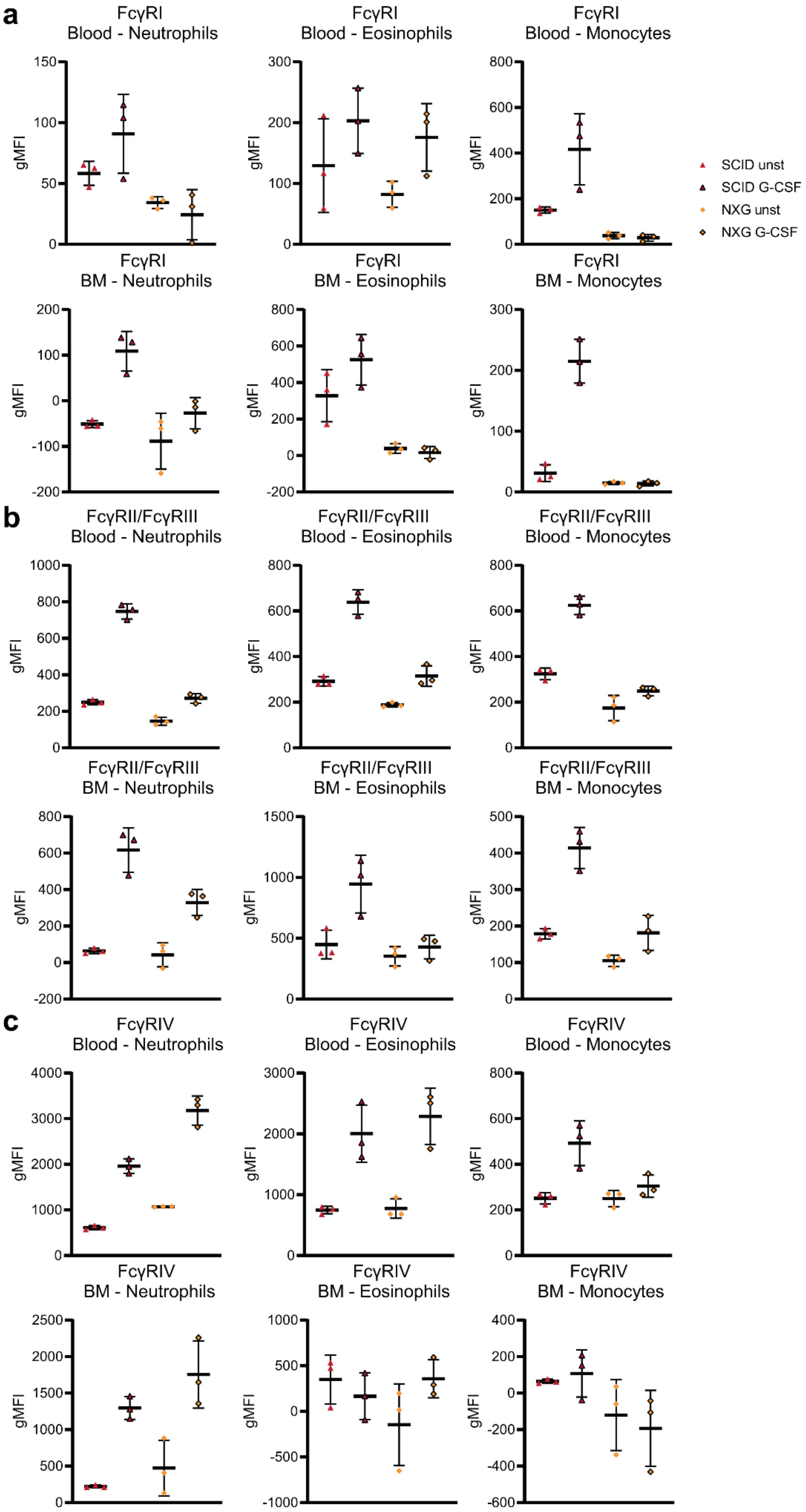

Fig. S4 FcγR expression in peripheral blood and bone marrow-derived myeloid cells Blood and bone marrow was obtained from either PEG-G-CSF or unstimulated male hCD89 TG mice from 4 mouse strains. Antibody staining and subsequent flow cytometry analyses were performed to determine expression of (**A**) FcγRI, (**B**) FcγRII/FcγRIII and (**C**) FcγRIV on neutrophils, eosinophils and monocytes. Unst = unstimulated.

Supplemental Figure 5

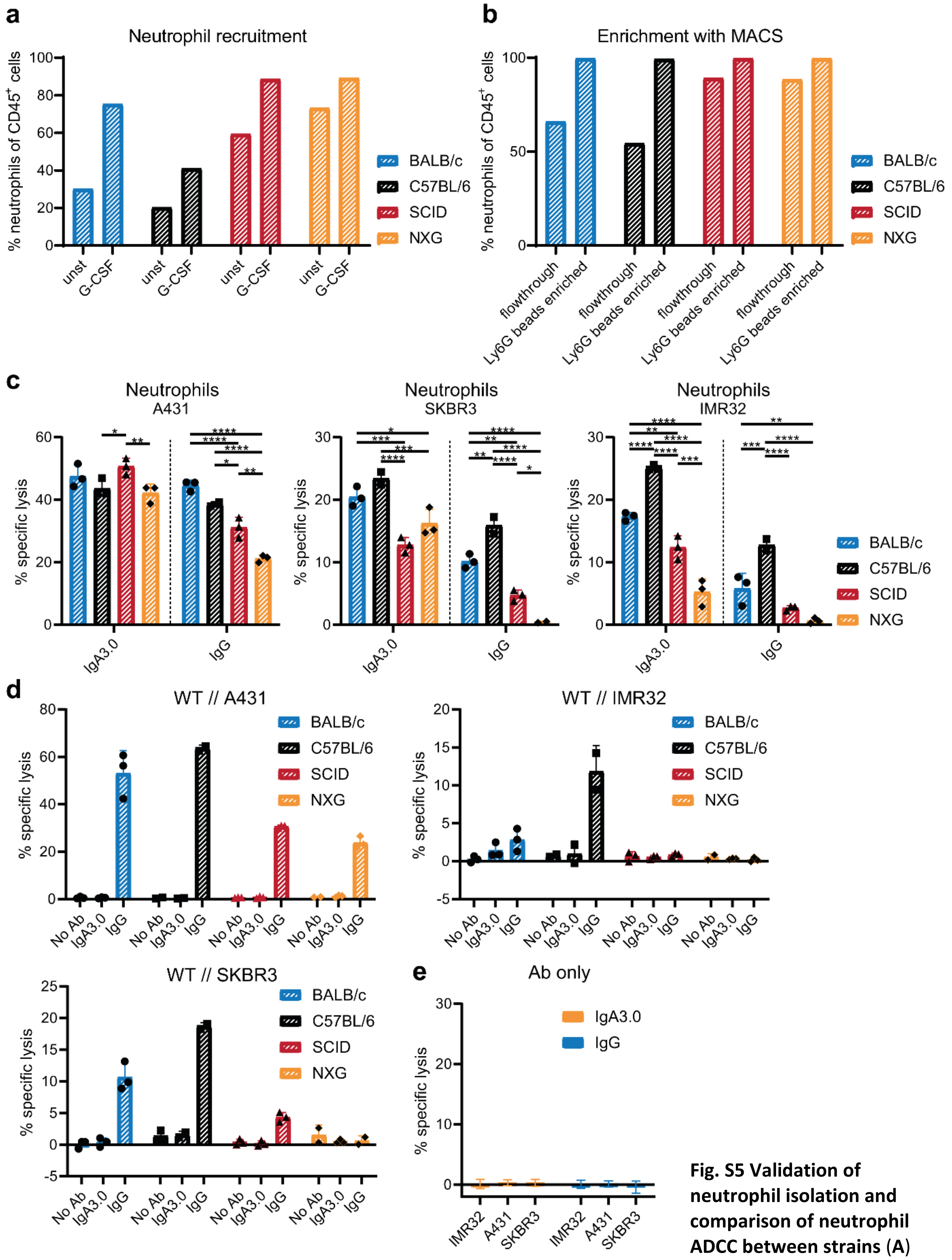

Fig. S5 Validation of neutrophil isolation and comparison of neutrophil ADCC between strains (**A**) Blood from PEG-G-CSF and unstimulated mice was collected and antibody staining and subsequent flow cytometry analyses were performed to determine the number of neutrophils in the circulation. (**B**) Cells in the flowthrough and enriched fraction after Ly6G MACS isolation were stained and analyzed by flow cytometry to validate neutrophil purity. (**C**) ^{51}Cr-release assays against 3 different targets/cell lines comparing IgA3.0- or IgG-mediated ADCC of isolated neutrophils from (**D**) 4 different hCD89 TG mouse strains and (**D**) WT mice. (**E**) IgA3.0 and IgG antibody only control in ^{51}Cr-release assays against 3 different targets/cell lines.

Supplemental Figure 6

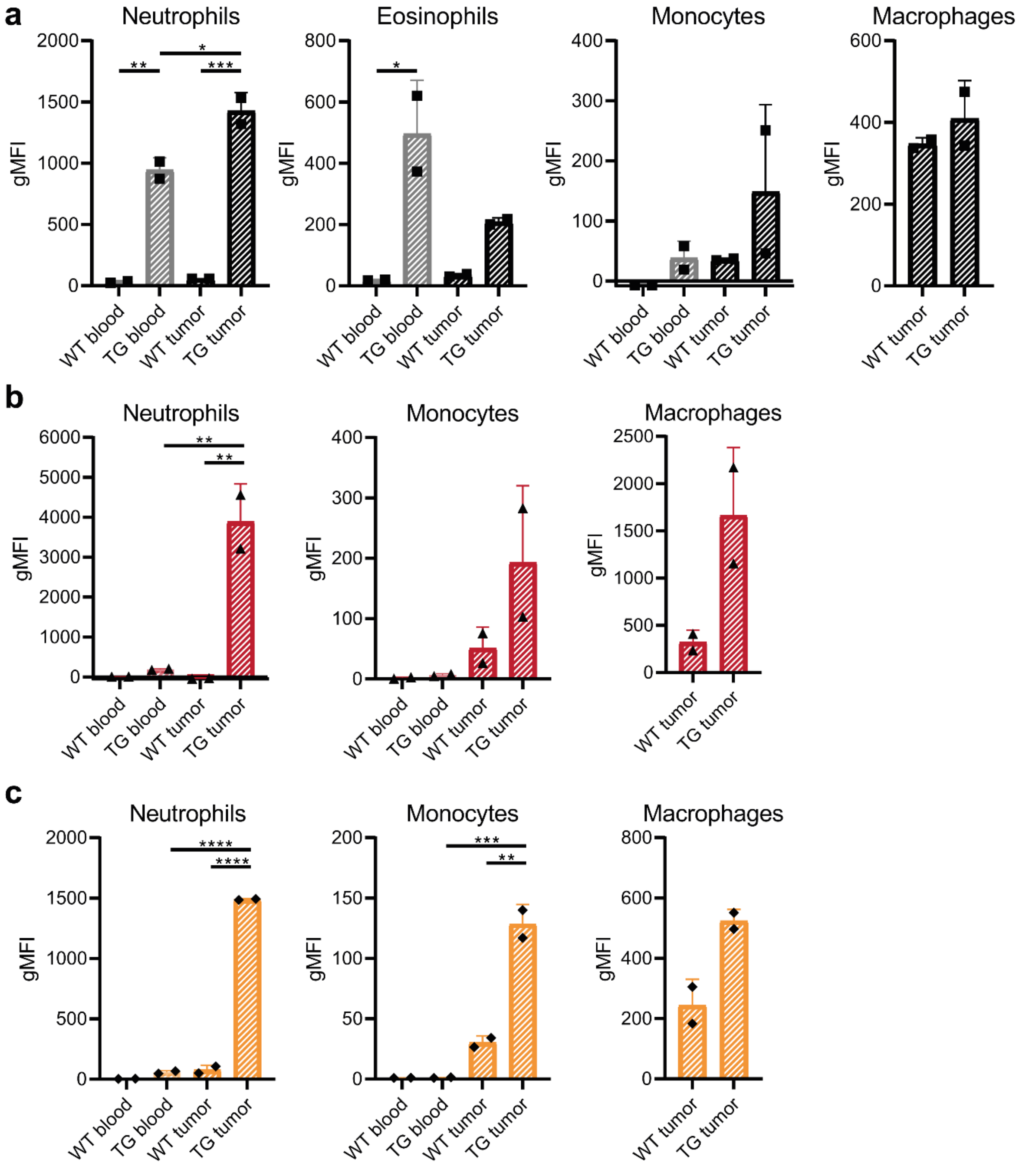

Fig. S6 CD89 expression on myeloid cells in the tumor microenvironment (**A**) Intraperitoneal 9464D-GD2 neuroblastoma tumors in C57BL/6 mice were established by injecting 0.5*10^6 9464D-GD2 cells intraperitoneally. CD89 expression on myeloid cell subsets in the circulation and tumor was determined on day 27 by flow cytometry. Subcutaneous IMR32 human xenograft tumors were established in both (**B**) SCID and (**C**) NXG mice by injecting 2.5*10^6 IMR32 cells subcutaneously. CD89 expression on myeloid cell subsets in the circulation and tumor was determined on day 51 by flow cytometry. Means of neutrophils, eosinophils and monocytes were compared using a One-Way ANOVA with Tukey's post-hoc test and means of macrophages were compared using an unpaired t-test. WT = wildtype, TG = transgenic.
